# Fetal Down syndrome screening models for developing countries; Part II: Cost-benefit analysis

**DOI:** 10.1186/s12913-019-4699-4

**Published:** 2019-11-27

**Authors:** Chanane Wanapirak, Piyaluk Buddhawongsa, Woraluck Himakalasa, Auttapan Sarnwong, Theera Tongsong

**Affiliations:** 1 Department of Obstetrics and Gynecology, Faculty of Medicine, Chiang Mai University, Chiang Mai, 50200 Thailand; 2 Faculty of Economics, Chiang Mai University, Chiang Mai, Thailand

**Keywords:** Down syndrome, Prenatal screening, Prenatal diagnosis, Cost-benefit, Developing country

## Abstract

**Background:**

To identify the most cost-beneficial model as a national policy of screening and diagnosis of fetal Down syndrome (DS) in developing countries.

**Methods:**

Cost-benefit analysis (CBA) was performed based on the effectiveness and probabilities derived from a large prospective study on MSS (maternal serum screening) among Thai population. Various models including maternal age alone, STS (second trimester screen), I-S (independent screen: first or second trimester screen depending on the time of first visit), C-S (contingent serum screen) plus STS, maternal age with NIPS (non-invasive prenatal test), STS alone with NIPS, I-S with NIPS, C-S plus STS with NIPS, and Universal NIPS were compared.

**Results:**

I-S with NIPS as a secondary screening was most cost-beneficial (Benefit/Cost ratio 4.28). Cost-benefit is directly related to the costs of NIPS.

**Conclusion:**

In addition to simplicity and feasibility, I-S with expensive NIPS as a secondary screening is the most cost-beneficial method for low resource settings and should be included in universal healthcare coverage as a national policy. This study could be a model for developing countries or a guideline for international health organizations to help low resource countries, probably leading to a paradigm shift in prenatal diagnosis of fetal DS in the developing world.

## Background

Prenatal screening and diagnosis of Down syndrome (DS) with maternal serum screening (MSS) has been established in developed countries [1]. However, the incidence in countries with low-resource settings has not changed much in recent years, in spite of the fact that there is more need for lower incidence in poorer countries, since DS can constitute more burdens due to the low quality of life in poor countries [2, 3]. Moreover, in low resource countries, the socio-economic gap is even wider. We believe that economic inequality results in health inequality. In this regard, the expensive but more effective non-invasive prenatal screen (NIPS) has been accepted by wealthy couples but the poor do not have the opportunity to benefit from it. The only way to overcome this problem is to include cost-benefit screening in universal health care coverage as a national policy. In Thailand, we are considering the implementation of DS screening for all women as a national policy free of charge. However, the most cost-beneficial model is not known, especially in developing countries. Additionally, the studies on the cost-benefit of such strategies are mostly based on simulations of hypothetical cohort instead of testing on real situations that are varied among geographical and racial groups. Many cost-benefit studies [4–7] relied on the western data of MSS developed for western population, but its efficacy is very different from that used in other parts of the world. Moreover, CBA (cost-benefit analysis) in previous studies were based on assumption that all women were assumed to attend prenatal care in the first trimester. This is not true in real practice. To yield the most reliable results of CBA, the input variables must be most accurate. Therefore, we conducted this CBA based on the effectiveness of various models of primary screenings and real probabilities of various events derived from our own large prospective project [8]. This study was done to determine the best model for developing countries based on feasibility, simplicity and cost-benefit, so as to be considered in the universal health care coverage. Accordingly, this study did not include the techniques that are not practicable in low resource settings, such as NT, which needs expertise and is not widely available; integrated MSS test, which needs two screenings and the costs are double. However, NIPS as a secondary screening test might be cost-beneficial even in poor countries if the primary screen (MSS) is very effective with a low false positive rate (a small number of amniocenteses). It is more feasible to make NIPS available all over a country than amniocentesis, with a large number of chromosome laboratories. Accordingly, we performed CBA for several strategies, both when combined with NIPS and when not combined, to identify the most cost-beneficial model as a national policy of screening and diagnosis of fetal Down syndrome (DS) in developing countries.

## Methods

This study was cost-benefit analysis (CBA) which was conducted with ethical approval by the institutional review board, as the second part of our previous study [8], which was prospectively conducted on 41,924 pregnancies in the northern part of Thailand, including 33 community hospitals. CBA was based on the effectiveness of MSS and probabilities of various events derived from the previous study [8] and was performed from both societal and government perspectives. Probabilities and costs were applied to a hypothetical cohort of 800,000 pregnant women, representing the estimated annual number of pregnancies in Thailand. This CBA was performed using decision tree for 10 models as follows: 1) Reference situation (Base case): No prenatal screening and no amniocentesis (invasive prenatal testing); 2) Maternal age alone: Advanced maternal age (≥ 35 years) was classified as high risk and was offered amniocentesis; 3) Maternal age with NIPS: Advanced maternal age (≥ 35 years) was classified as high risk and offered NIPS. If NIPS was positive, amniocentesis was offered; 4) STS (second trimester screening) alone: Screening all women in the second trimester (15–20 weeks of gestation) and amniocentesis was offered in case of high risk serum markers; 5) STS with NIPS: Screening all women in the second trimester and NIPS was offered in case of high risk serum markers and amniocentesis was performed if NIPS was positive; 6) Independent screen (I-S): Women seen in the first trimester (9–14 weeks) were screened with FTS (first trimester screening) and those seen in the second trimester (15–20 weeks) were screened with STS. The women at high risk either by FTS or STS were offered amniocentesis; 7) I-S with NIPS: The same as no.6; but the high risk cases either by FTS or STS were offered NIPS and then amniocentesis in case of positive NIPS; 8) C-S (contingent serum screen) plus STS: Women seen in the first trimester (9–14 weeks) were screened with FTS and were classified as high risk (risk > 1:30) indicated for amniocentesis, intermediate risk (risk between 1:30–1:1500) indicated for STS and reclassified risk into low or high risk (> 1:250) by all serum markers, and low risk (risk < 1500) needed no further tests. The women seen in the second trimester (15–20 weeks) were screened with STS mentioned above (no. 4); 9) C-S plus STS, with NIPS: The same as no. 8; but the high risk cases were offered NIPS instead and amniocentesis in cases of positive NIPS; 10) Universal NIPS: All pregnant women before 20 weeks of gestation were offered NIPS and amniocentesis in cases of positive NIPS.

**Fig. 1:**
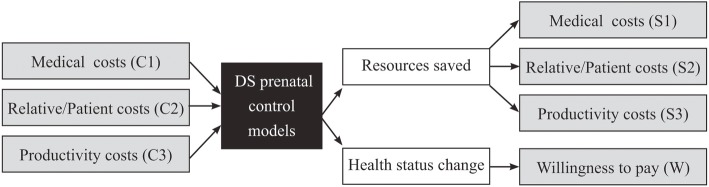
Components of economic calculation of Down syndrome (DS) control models

The CBA was based on the concept in Fig. 1 which compared the costs and outcomes of the models in money units. The costs included medical cost, family and relative costs and productivity (C1 + C2 + C3). They also included a number of DS secondary to false negative of the screening tests, non-acceptance of pregnancy termination of fetal DS and productivity of normal fetuses ending-up with fetal loss caused by amniocentesis. The benefits of the model included the sum of willingness to pay money to avoid having a DS baby and costs saved from the avoidance of DS (S1 + S2 + S3: direct and indirect life time costs and productivity). Cost-benefit calculation was expressed as incremental benefit-to-cost ratio (∆ benefit / ∆ cost) and incremental benefit-to-cost difference (∆ benefit – ∆ cost), whereas ∆ benefit is the benefit of any situation minus the benefit in the reference situation or without any screening and ∆ cost is the cost of any situation minus the cost in the reference situation.

Direct medical cost included screening tests and prenatal diagnosis (serum markers, cytogenetic lab, counseling and termination of pregnancy) and cost of taking care of DS child (physical program, treatment of complications, stimulating/promoting development). Direct non-medical cost included accommodation, meals, commuting of the patients and relatives during doctor visits and informal care of DS child. Indirect cost included productivity of: 1) the patients and relatives during doctor visits, 2) normal child terminated due to false positive tests, and 3) the relatives of taking care of the DS child. Costs from societal perspective included all items mentioned above while costs from governmental perspective included only direct medical cost. Probabilities of events and variables in effectiveness of screening tests and prenatal diagnoses: sensitivities and specificities (using risk cut-off 1:250) were derived from the study in the same population [8]. The costs of screening and prenatal diagnosis were assessed from our centers with micro-costing analysis. Direct and indirect costs of taking care of DS were derived from well-established publications [2, 9–11], with conversion to be equivalent to the Thai costs of living. All costs were adjusted to the costs in 2015 with consumer price index (CPI). The benefits consisting of the costs saved by the avoidance of new cases of DS and benefits in health outcomes were assessed from “willingness to pay (WTP) survey”. WTP was based on the interview (in Thai language) of women aged 25–45 years, in the year 2014 with the following question. “In the scenario that you are pregnant and your baby is affected by Down syndrome, what is the highest payment you are willing to make to avert the scenario? The payment you have to make once in six months. This is in the condition that you are willing to give birth to a Down’s baby and the government is responsible for all costs of living such as cost of taking care, illness treatment, nursing etc.” Then the willingness-to-pay was evaluated with bidding or bargaining format. In this study the interviewer offered the starting point of 5000 to 300,000 Thai baht to reduce biases which might occur during bargaining. The uncertainty analysis was performed using one-way and probabilistic sensitivity analyses. The CBA was analyzed using A software package (TreeAge Pro 2009, TreeAge Inc., Williams-town, MA, USA).

## Results

We based the CBA on 41,924 screens with a risk cutoff of 1:250, both the model performance and event probabilities as summarized in Tables 1 and 2. The accuracy of NIPS (99.0%) was based on previous studies [12] and the acceptance rate (99.0%, in case of free of charge) was assumed based on the acceptance of MSS in our previous study [8]. Costs screening tests used in the CBA were calculated by micro-costing, representing costs in Thailand in the year 2015. However, the life-time cost for taking care of a DS child among Thai population with reliable and comprehensive analysis was not available. Thus, we used the cost from the western well-established data with being converted to cost equivalent to Thai cost of living using purchasing power parity conversion factor (Consumer Price Index of THAILAND YEAR 2019; conversion factor LCU per international $ = 13.04). The cost of NIPS was based on Thai NIPS which was the lowest price in Thailand in 2019. The willingness to pay (WTP) to avert having DS child in women of all age was based on the survey study among Thai population. Rates of acceptance were based on the assumption that various prenatal screening tests or diagnostic tests were free of charge, according to health coverage by the national policy. The probabilities of various events and cost used in the CBA are summarized and presented in Table 1, 2 and 3. All models for CBA were based on Thai reference range (TRR). Probabilities and costs were applied to a hypothetical cohort of 800,000 pregnant women, representing the estimated annual number of pregnancies in Thailand. This CBA was performed using decision tree for 10 models as stated in the “Methods” section.

**Table 1:** Probabilities of the events used in the simulations [8]

Variables	Distri-bution	Mean
Prevalence of women attending antenatal care in the first trimester	normal	0.7080
Prevalence of DS at 16 weeks of gestation in women of < 35 years of age	beta	0.0016
Prevalence of DS at 16 weeks of gestation in women of ≥ 35 years of age	beta	0.0057
Prevalence of DS at 16 weeks of gestation in women of all age	beta	0.0018
Prevalence of DS in women of all age	beta	0.0018
MSS uptake among women of all age	beta	0.9557
Amniocetesis uptake among women of all age	beta	0.9245
Spontaneous abortion of DS fetuses at 10 weeks of gestation in women of all age	beta	0.2713
Spontaneous abortion of DS fetuses at 16 weeks of gestation in women of all ages	beta	0.2175
Spontaneous abortion of DS fetuses in women of all age	beta	0.2920
Termination of pregnancy in case of +ve amniocentesis	normal	0.9500
Amniocentesis-related fetal loss	beta	0.0050
NIPS uptake (assumption for free of charge)	beta	0.9900

**Table 2:** Sensitivity and specificity of prenatal screening test and diagnostic test used in the simulations [8]

Screening / Diagnostic Tests	Type of distribution	Sensitivity	Specificity
FTS	beta	0.792	0.932
STS (Triple screen)	beta	0.762	0.908
Independent screen (I-S)	beta	0.784	0.925
Contingent screen (C-S plus STS)	beta	0.849	0.923
NIPS [12]	beta	0.990	0.980

**Table 3:** Costs used in the simulations (expressed in USD, adjusted value for the year 2019)

Costs	Type of distribution	Cost from government perspectives (direct medical)	Direct non-medical cost of a woman and relatives	Indirect cost	Cost from societal perspective	References
FTS (first trimester screen)	gamma	30.63	58.02		88.65	Calculated by micro-costing
STS (second trimester screen)	gamma	33.65	58.02		91.67	
Amniocentesis and chromosome study & counseling	gamma	141.56	58.02		199.58	
Intended termination of pregnancy	gamma	77.31	–		77.31	
Vaginal delivery	gamma	66.50	–		66.50	
Cesarean delivery	gamma	245.64	–		245.64	
NIPS	gamma	416.86	58.02		474.88	Thai NIPS
Lifetime costs of taking care of DS	gamma	103,251.46		479,892.65	583,144	Ref [2, 9–11]^a^
Indirect costs of normal child (in case of termination due to false positive)	gamma			44,229	44,229	Ref [3]
WTP to avert having DS child in women of all age	gamma	1945			1945	Questionnaire

^a^ This study transfers the lifetime costs of Down syndrome children from the previous studies to measure the medical costs (ref 11) and indirect lifetime costs. Since the information from those studies was based on samples in the United States and the studies were conducted in 2011 and 2017, the value of transferred cost applied in this study needs to be adjusted according to Thailand context and the time of valuation. The purchasing power and currency adjustment between Thai and the U.S. is adjusted by the PPP conversion factor (World bank, 2019: PPP conversion factor, private consumption (LCU per international $) Retrieved October 1, 2019, from http://data.worldbank.org/indicator/PA.NUS.PRVT.PP) and the different time period is adjusted by Consumer Price Index (CPI) (Ministry of Commerce, 2019: Consumer Price Index of THAILAND YEAR 2019 BASE YEAR 2011 and 2017. Retrieved October 1, 2019, from http://www.indexpr.moc.go.th/price_present/TableIndexG_region.asp?table_name=cpig_index_country&province_code=5&type_code=g&check_f=i&year_base=2560&nyear=2562 AND Bank of Thailand (2019) Historical Foreign Exchange Rates Retrieved October 1, 2019, from https://www.bot.or.th/english/_layouts/application/exchangerate/exchangerateago.aspx)

**Fig. 2:**
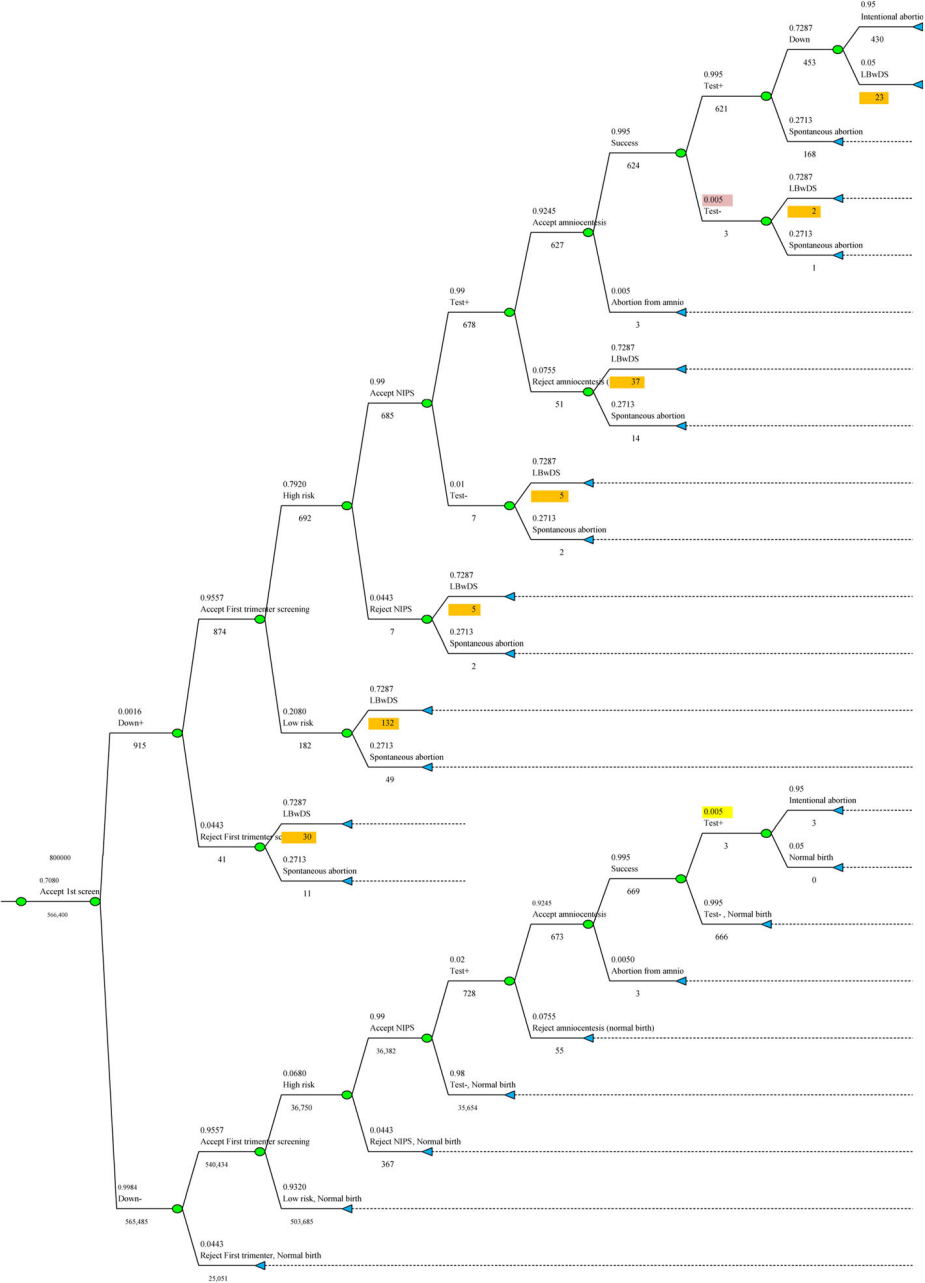
An example of decision tree, FTS as a part of I-S with NIPS (Model 8), shows probability and numbers of DS and non-DS group at each step of screening which could be detected or missed from the first step of acceptance/rejection of screening through various steps to definite diagnosis. The number of cases at each step was used for cost calculation. At first step, 70.8% of 800,000 women underwent FTS with NIPS (nearly 30% had no screen; including some cases with DS), consisting of 915 DS and 565,485 non-DS pregnancies. Each group had branching for a further series of actions in case of positivity. For example, DS group consisted of high risk and low risk pregnancies. The low risk had no further test, though consisting of live birth DS and DS with spontaneous abortion. Nearly all high risk pregnancies accepted but some rejected NIPS; nearly all cases with +ve NIPS accepted amniocentesis in which nearly all cases had successful procedures and few had failed procedure or lab failure). Failure to detect DS could occur at any cascade; and finally not all detected DS accepted intentional abortion. Likewise, of non-DS group, false positive result could occur at any cascade, though it should not (lab error, specimen switching); intentional abortion could also occur though very rare

**Fig. 3:**
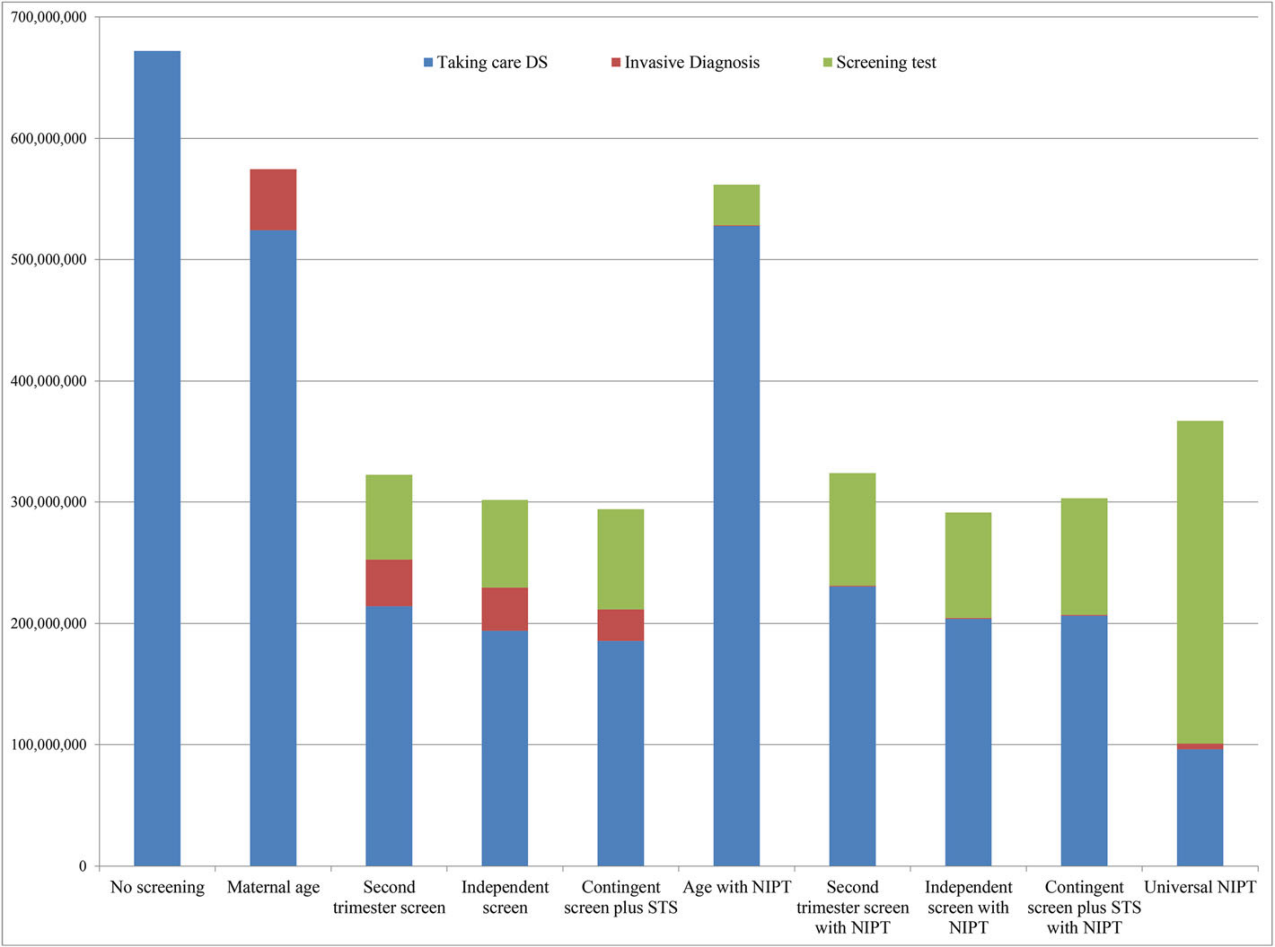
Cost for each model in prenatal control of fetal Down syndrome among 800,000 pregnancies (societal perspective)

The CBA used decision-analytic modeling as an example in Fig. 2, to determine the outcomes, total costs, relative costs, cost-benefit difference and ratio, and events of different models are presented in Table 3 and 4. It directly compares current clinical practice in most parts of Thailand, no screening as the base case. From societal perspective, I-S with NIPS would be most cost-beneficial when the cost of NIPS $416.86 or less, giving B/C ratio of 4.28. If NIPS is more expensive, C-S plus STS (without NIPS) would be most cost-beneficial (Table 5, Fig. 3). However, its detection rate was slightly lower when compared with the C-S plus STS model. The most cost-beneficial model, from governmental perspective, was the independent screening without NIPS, giving B/C ratio of 2.30. Cost-benefit is directly related to the costs of NIPS (Table 6). I-S with NIPS gave the B/C ratio of 4.84 if the cost of NIPS was decreased to $277 (This is Thai NIPS cost in 2019).

**Table 4:** Expected events in various situations of 800,000 pregnant women in one year (see an example of decision tree for model in Fig. 2)

Situations	No. DS live births	No. of spon-taneous abortion	No. of intention termina-tion	No. of abortion due to amniocenteses		No. of amniocen-teses and chromosome studies
				Down + ve	Down -ve	
1. No screening	1152	475	–	–	–	–
2. Maternal age alone	899	491	705	2	428	86,158
3. Second trimester screen (STS) alone	367	280	947	4	325	65,794
4. Independent screen (I-S)	333	329	876	4	265	53,827
First trimester screen (FTS)	*226*	*247*	*600*	*3*	*136*	*34,615*
STS	*107*	*82*	*277*	*1*	*78*	*19,212*
5. C-S plus STS	318	296	841	5	178	36,592
*C-S*	*211*	*214*	*565*	*3*	*67*	*17,380*
*STS*	*107*	*82*	*277*	*1*	*76*	*19,212*
6. Maternal age with NIPS	905	489	302	2	8	2178
7. STS alone with NIPS	395	284	633	4	6	2093
8. Independent screen (I-S) with NIPS	350	330	618	4	5	1911
*FTS with NIPS*	*234*	*247*	*433*	*3*	*3*	*1300*
*STS with NIPS*	*115*	*83*	*185*	*1*	*2*	*611*
9. C-S plus STS with NIPS	354	323	619	4	3	1560
*C-S + NIPS*	*239*	*240*	*434*	*3*	*2*	*949*
*STS + NIPS*	*115*	*83*	*185*	*1*	*1*	*611*
10. Universal NIPS	165	473	1051	7	73	16,089

**Table 5:** Costs and benefits (USD/woman) of various models from societal and government perspective when cost of NIPS is $416.86 (13,000 THB)

Strategies	Societal Perspective					Government Perspective				
	Cost of screening and prenatal diagnosis (1)	Cost saved by avoiding DS births (2)	WTP (3)	Benefit-cost difference (4)	Benefit- cost ratio (5)	Cost of screening and prenatal diagnosis (1)	Cost saved by avoiding DS births (2)	WTP (3)	Benefit-cost difference (4)	Benefit- cost ratio (5)
1. No screening	0	0	0	0	0	0	0	0	0	0
2. Maternal age alone	80.0	184.5	0.7	105.2	2.32	18.1	32.7	0.7	15.3	1.84
3. STS alone	164.4	571.9	2.3	409.7	3.49	51.9	101.3	2.3	51.6	1.99
4. Independent screen (I-S)	150.9	597.1	2.4	448.6	3.97	47.0	105.7	2.4	61.1	2.30
5.C-S plus STS	149.4	607.6	2.4	460.6	4.08	48.8	107.6	2.4	61.2	2.26
6. Maternal age with NIPS	62.2	180.1	0.7	118.7	2.91	52.4	31.9	0.7	−19.8	0.62
7. STS alone with NIPS	146.4	551.9	2.2	407.7	3.78	74.0	97.7	2.2	25.9	1.35
8. I-S with NIPS	137.1	584.9	2.3	450.1	4.28	65.8	103.6	2.3	40.1	1.61
9. C-S plus STS with NIPS	139.4	581.5	2.3	444.4	4.19	60.8	103.0	2.3	44.5	1.73
10. Universal NIPS	494.8	719.4	2.8	227.4	1.46	416.2	127.4	2.8	− 286.0	0.31

Benefit-cost difference = [(2 + 3) _any situation_ – (2 + 3) _situation 1_] – [1 _any situation_ – 1 _situation 1_]

Benefit-cost ratio = [(2 + 3) _any situation_ – (2 + 3) _situation 1_] / [1 _any situation_ – 1 _situation 1_]

**Table 6:** Benefits costs ratios (B/C) of various strategies from societal and government perspective at different costs of NIPS (Cost of Thai NIPS $416.86 in 2016 and $277.90 in 2019)

Strategies	Societal Perspective					Government Perspective				
	$833.71	$694.76	$555.81	$416.86	$277.90	$833.71	$694.76	$555.81	$416.86	$277.90
1. No screening	0	0	0	0	0	0	0	0	0	0
2. Maternal age alone	2.32	2.32	2.32	2.32	2.32	1.84	1.84	1.84	1.84	1.84
3. STS alone	3.49	3.49	3.49	3.49	3.49	1.99	1.99	1.99	1.99	1.99
4. Independent screen (I-S)	3.97	3.97	3.97	3.97	3.97	2.30	2.30	2.30	2.30	2.30
5. C-S plus STS	4.08	4.08	4.08	4.08	4.08	2.26	2.26	2.26	2.26	2.26
6. Maternal age with NIPS	1.59	1.87	2.28	2.91	4.24	0.31	0.38	0.47	0.62	1.32
7. STS alone with NIPS	3.05	3.26	3.50	3.78	4.35	0.91	1.02	1.16	1.35	1.81
8. I-S with NIPS	3.52	3.74	4.00	4.28	4.84	1.11	1.24	1.40	1.61	2.12
9. C-S plus STS with NIPS	3.68	3.83	4.00	4.19	4.52	1.31	1.43	1.56	1.73	2.09
10. Universal NIPS	0.80	0.94	1.14	1.46	2.63	0.16	0.19	0.24	0.31	0.66

If 60, 50 and 40% of women first present in the first trimester, sensitivity analysis for societal perspective still shows that I-S with NIPS is most cost-beneficial with B/C ratio of 4.15, 3.87 and 3.59, respectively. (not shown in Table).

## Discussion

The important insights gained from this study are: 1) While C-S was the most effective serum screening test, the most cost-beneficial model, from societal perspective, was I-S with NIPS, though the detection rate was slightly lower than C-S plus STS model. 2) The most cost-beneficial model from governmental perspective was I-S, without NIPS. Nevertheless, as a national policy, CBA is better based on societal perspective since taking care of DS child both direct and indirect costs as well as productivity loss due to amniocentesis are all societal burdens. 3) Cost-benefit is directly related to the costs of NIPS. In addition, I-S with NIPS is also the most practical model, in terms of patient’s convenience of first visit timing and only once screening.

To be most accurate in cost-benefit analysis, the input values must be reliable. Accordingly, we used most reference ranges derived from our own population, in a large prospective study, since several parameters varies among geographical areas because of racial / biophysical factor and body size. As seen in the part I of the same project as this study, screening performance in detecting Down syndrome is significantly different between serum markers based on Caucasian reference range and Thai reference range; for example, false positive rate of 13.7% vs 7.7%, respectively, for contingent screen. Therefore, this new study (part II of the same project [8]) used various input values derived from our own reference ranges. Likewise, though natural pregnancy loss is also slightly different from the western studies [13], we preferred using our own data.

Current clinical practice in obstetrics has shifted the paradigm from a conventional prenatal approach based on invasive procedures, to non-invasive prenatal testing for some fetal aneuploidies via NIPS. Since the rapid spread around the world of prenatal diagnosis based on NIPS, it is time to start thinking how this cutting-edge technology might influence current practice of obstetrics in low-resource countries since NIPS will become available in low-resource countries in the foreseeable future [14]. Therefore, we included NIPS in CBA model in this study. Most developing countries have acute limitation of chromosome laboratory and no DS screening, though some countries have DS screening, mostly based on advanced maternal age. MSS is rarely available in the public sector. The main problem is that most poor women cannot access this kind of health care service which is usually available only in the private sector. Only rich couples undergo MSS and NIPS. In near future, DS will be a genetic condition of higher prevalence among those of lower socioeconomics means in comparison to their counterparts with higher socioeconomic means. Certainly, in developing countries, the percentages of NIPS uptake and serum screening are expected to be low, despite the fact that such models are more cost-beneficial than lack of screening or age-based screening as suggested.

Considering the best model for developing countries, several aspects must be taken into account: feasibility, expertise requirement, simplicity, costs of screening tests and invasive diagnosis, capacity in chromosome lab development etc. Note that this study did not include integrated tests, because of the high costs of double screenings with small additional detection rate. It also excluded NT and genetic sonogram, because of the need for high expertise, not practical in low resource settings. FTS alone was not suitable since many women had their first visit in late gestation. C-S plus STS was most effective but had higher costs due to the high rate of intermediate risk requiring STS and was complicated by counseling as well as anxiety during waiting for the final risk. Therefore I-S seems to be more attractive, though with slightly lower detection rate. Moreover, our findings surprisingly indicate that, even in low resource settings, incorporation of the expensive NIPS as a secondary test for high risk women is cost-beneficial, though NIPS as a primary screening is not cost-beneficial and not suitable for developing countries, unless its cost is markedly reduced. Challengingly, the poorer the country, the higher the need for the availability of NIPS, instead of karyotype laboratories. For example, in Thailand, we may need only one effective NIPS center to serve the entire country, whereas we may need a hundred cytogenetic laboratories, including intensive training of more than 1000 technicians to cover the screening of all the 800,000 Thai pregnant women each year. To date, our country could not perform chromosome studies more than 20,000 cases per year. Therefore, NIPS as a secondary screening is more feasible and more cost-effective to serve an entire country, without the overload of amniocentesis and chromosome laboratories, especially when the cost of NIPS reduces. However, although the CBA derived from this study may not be applied to many other countries with lower or higher resource setting than Thailand, it can serve as a study model for other countries.

Because health care resources are limited, CBA-based modeling must be used to guide resource allocation. Since the current practice using conventional or age-based screening would be the least costly model, decision-makers might tend to refrain from implementing NIPS in national health care. However, our CBA indicates that the benefits of NIPS should not be underestimated. In addition to high accuracy, ease to understand, and safe option, the need for less number of experts and chromosome labs must be taken into account. Its implementation could directly facilitate the ultimate goal of the national program for prenatal control of fetal DS. The cost-benefit of NIPS is directly related to its costs and the estimated costs of taking care of a life time of DS. Thus, investments in NIPS would in fact be outweighed by a concurrent decrease in health care and societal costs associated with DS. Note that if we use Caucasian reference ranges of MSS, the false positive rate is very high, leading to a substantial burden of invasive diagnosis as well as fetal loss or expensive NIPS and possibly no cost-benefit. Such effects may not be so serious for the payer perspective but are very serious for societal and government perspectives. Therefore, we strongly recommend the development of the normal reference ranges of the intended population for the formation of a national policy.

The strengths of this study are as follows: 1) CBA was based on the strategy effectiveness data and event probabilities derived from the same population and real situations as well as consideration of the proportion of women with late visits of prenatal care. 2) The costs of all tests and medical or non-medical care based on real situations of developing countries like Thailand.

The weaknesses of this study are as follows: 1) CBA did not include payer perspective. 2) The structures and inputs of the decision-analytic model in this study were primarily focused on our national health care. Thus, the results might not be perfectly accurate for other countries’ strategies. However, we believe that this could probably be a model for several developing countries especially many parts of Asia. 3) Though I-S with NIPS is most cost-beneficial, its true feasibility of implementation has not yet been explored. 4) Our CBA was derived from Thailand, these data might not perfectly be translated to other developing countries due to uncertainties in uptake or variations in the purchasing power and currency adjustment (World Bank data). Therefore, our results must be cautiously interpreted when applied for other developing countries. 5) This study focused exclusively on DS, did not address the cost issues of the bigger picture. On economic view, DS is only a small part of complete chromosomal and copy number variant abnormalities that can produce as much personal and financial burdens to any population. 6) Indirect cost of taking care of DS might not be perfectly validated. For example, incremental cost savings due to less chance of DS patients to attend university or get married, among many other potential social factors were not incorporated in analysis. As such, our analyses might be positively skewed toward the benefits of prenatal screening.

## Conclusion

1) The challenging finding is that, even in low resource settings, I-S with NIPS seems to be most cost-beneficial. Women of all socio-economic levels should have an equal chance to access this facility. 2) The strong impact factors of cost-benefit include cost of NIPS, cost of taking care of a DS child and false positive rate of MSS. 3) Our findings emphasize that the most expensive public policy is to have no screening. Additionally, cost-benefit can be much varied at different NIPS costs. It can change enormously based on changing costs of individual elements, miscalculations in percentage of choices people make, etc.

## Data Availability

Data is available through the corresponding author. The data set is filed at the Chiang Mai University Thailand. The data set has not been deposited in a public repository due to the confidential nature of patient data and issues with confidentially and anonymity in the small, rural community we were working in.

## Abbreviations

CBA: Cost-benefit analysis
CPI: Consumer price index
C-S: Contingent first trimester screening
DS: Down syndrome screening
FTS: First trimester screening
I-S: Independent first and second trimester screening
MSS: Maternal serum screening
NIPS: Non-invasive prenatal test
STS: Second trimester screening
WTP: Willingness to pay
